# 2763. Evaluating the Safety and Effectiveness of Long-term Antibiotic Suppression in Patients with Left Ventricular Assist Devices

**DOI:** 10.1093/ofid/ofad500.2374

**Published:** 2023-11-27

**Authors:** Kelsey D Hamlin, P Brandon Bookstaver, Caroline Derrick, Chengwen Teng, Jacob Brown, Andrew Skweres, Andrew Mardis, Laura Straw, Julie Ann Justo, James Ampadu, Majdi N Al-Hasan, Kamla Sanasi, Hana R Winders

**Affiliations:** Prisma Health Richland, Columbia, South Carolina; Prisma Health Richland - University of South Carolina, Columbia, South Carolina; University of South Carolina School of Medicine, Columbia, South Carolina; University of South Carolina School of Pharmacy, Columbia, South Carolina; University of South Carolina School of Pharmacy, Columbia, South Carolina; University of South Carolina School of Pharmacy, Columbia, South Carolina; Prisma Health Heart Hospital, Columbia, SC; Prisma Health Richland, Columbia, South Carolina; Dartmouth Hitchcock Medical Center; Prisma Health Richland, Columbia, South Carolina; University of South Carolina School of Medicine, Columbia, South Carolina; Prisma Health Richland, Columbia, South Carolina; Prisma Health Richland, Columbia, South Carolina

## Abstract

**Background:**

The utilization of left ventricular assist devices (LVADs) as a bridge to transplantation or destination therapy for non-transplant candidates continues to rise. Unfortunately, infection remains one of the most common complication and lack of definitive source control often leads to the decision to initiate long-term antibiotic suppression. The purpose of this retrospective cohort study is to evaluate the long-term safety and effectiveness of antibiotic suppression following an LVAD-related or LVAD-specific infection.

**Methods:**

Adults who underwent LVAD placement between August 2018 and September 2022 at Prisma Health Midlands and received antibiotic suppression for an LVAD-related or LVAD-specific infection were evaluated. Safety was evaluated as a composite endpoint of treatment emergent adverse events, and discontinuation of or decrease in dose due to tolerability concerns. Effectiveness was evaluated as the proportion of patients without a suppression emergent infection (SEI) within six months of antibiotic suppression initiation. Patients experiencing a SEI (failure) were compared to those without an SEI (success) at six months. Secondary outcomes included the proportion of patients experiencing early SEI within 90 days of antibiotic suppression initiation and evaluation of factors associated with success of antibiotic suppression at six months.

**Results:**

Fifty-one patients received antibiotic suppressive therapy following an LVAD-related or LVAD-specific index infection. Patients were followed for a minimum of 6 months and an average 2.8 years. Treatment emergent adverse events occurred in 4 (7.8%) patients, with 2 events leading to antibiotic discontinuation. SEI occurred in 9 (17.6%) of patients at 6 months with the most common organisms being *P*. *aeruginosa*, *S. aureus*, and *S. epidermidis*. Early SEI was observed in 4 (7.8%) patients. Overall, SEI occurred in 28 patients (55%) at a median 341 days (IQR 157-502). Documented resistance to suppressive antibiotic was observed in 13 (25.4%) patients. Index infections with a gram-negative organism was significantly more common in those experiencing SEI (80% vs. 47%, p=0.031).

**Conclusion:**

Antibiotic suppression following LVAD-associated infections is well-tolerated and successful in 82% of patients at 6 months.

**Disclosures:**

**Julie Ann Justo, PharmD, MS, FIDSA, BCPS**, Gilead Sciences: Advisor/Consultant|Shionogi: Advisor/Consultant|Vaxart: Stocks/Bonds

